# Correlations of typical pain patterns with SPECT/CT findings in unhappy patients after total knee arthroplasty

**DOI:** 10.1007/s00167-021-06567-y

**Published:** 2021-04-17

**Authors:** Dominic T. Mathis, Samuel Tschudi, Felix Amsler, Antonia Hauser, Helmut Rasch, Michael T. Hirschmann

**Affiliations:** 1 University of Basel, 4051 Basel, Switzerland; 2 Department of Orthopaedic Surgery and Traumatology, Kantonsspital Baselland (Bruderholz, Liestal, Laufen), 4101 Bruderholz, Switzerland; 3 Amsler Consulting, 4059 Basel, Switzerland; 4 Institute of Radiology and Nuclear Medicine, Kantonsspital Baselland (Bruderholz, Liestal, Laufen), 4101 Bruderholz, Switzerland

**Keywords:** Pain, Character, Total knee arthroplasty, Pattern, Pathology, SPECT/CT, Position, Total knee replacement

## Abstract

**Purpose:**

The diagnostic process in patients after painful total knee arthroplasty (TKA) is challenging. The more clinical and radiological information about a patient with pain after TKA is included in the assessment, the more reliable and sustainable the advice regarding TKA revision can be. The primary aim was to investigate the position of TKA components and evaluate bone tracer uptake (BTU) using pre-revision SPECT/CT and correlate these findings with previously published pain patterns in painful patients after TKA.

**Methods:**

A prospectively collected cohort of 83 painful primary TKA patients was retrospectively evaluated. All patients followed a standardized diagnostic algorithm including 99m-Tc-HDP-SPECT/CT, which led to a diagnosis indicating revision surgery. Pain character, location, dynamics and radiation were systematically assessed as well as TKA component position in 3D-CT. BTU was anatomically localized and quantified using a validated localization scheme. Component positioning and BTU were correlated with pain characteristics using non-parametric Spearman correlations (*p* < 0.05).

**Results:**

Based on Spearman’s rho, significant correlations were found between pain and patients characteristics and SPECT/CT findings resulting in nine specific patterns. The most outstanding ones include: Pattern 1: More flexion in the femoral component correlated with tender/splitting pain and patella-related pathologies. Pattern 3: More varus in the femoral component correlated with dull/heavy and tingling/stinging pain during descending stairs, unloading and long sitting in patients with high BMI and unresurfaced patella. Pattern 6: More posterior slope in the tibial component correlated with constant pain.

**Conclusion:**

The results of this study help to place component positioning in the overall context of the "painful knee arthroplasty" including specific pain patterns. The findings further differentiate the clinical picture of a painful TKA. Knowing these patterns enables a prediction of the cause of the pain to be made as early as possible in the diagnostic process before the state of pain becomes chronic.

**Level of evidence:**

Level III

**Supplementary Information:**

The online version contains supplementary material available at 10.1007/s00167-021-06567-y.

## Introduction

The causes for recurrent pain after total knee arthroplasty (TKA) are manifold and range from knee joint-related factors such as infection, arthrofibrosis, patellofemoral problems, malposition or malalignment, loosening or instability to non-knee joint-related causes such as psychological disorders, vascular pathologies, back or hip problems [5, 23].

The diagnostic process is challenging. Besides a detailed patient history, a thorough clinical examination, radiological, serological and microbiological investigations are part of a standardized diagnostic algorithm for unhappy patients after TKA [23, 35]. As radiological work-up conventional radiographs, stress radiographs, CT or single-photon-emission-computed-tomography/computer tomography (SPECT/CT) and magnetic-resonance imaging (MRI) are often indicated [10, 16]. After a thorough diagnostic work-up, a revision surgery should only be performed if the cause(s) of the complaints described were identified and fit the clinical picture. Revision surgery for unexplained pain has consistently been shown to result in poor outcomes [16, 23, 30].

**Fig. 1:**
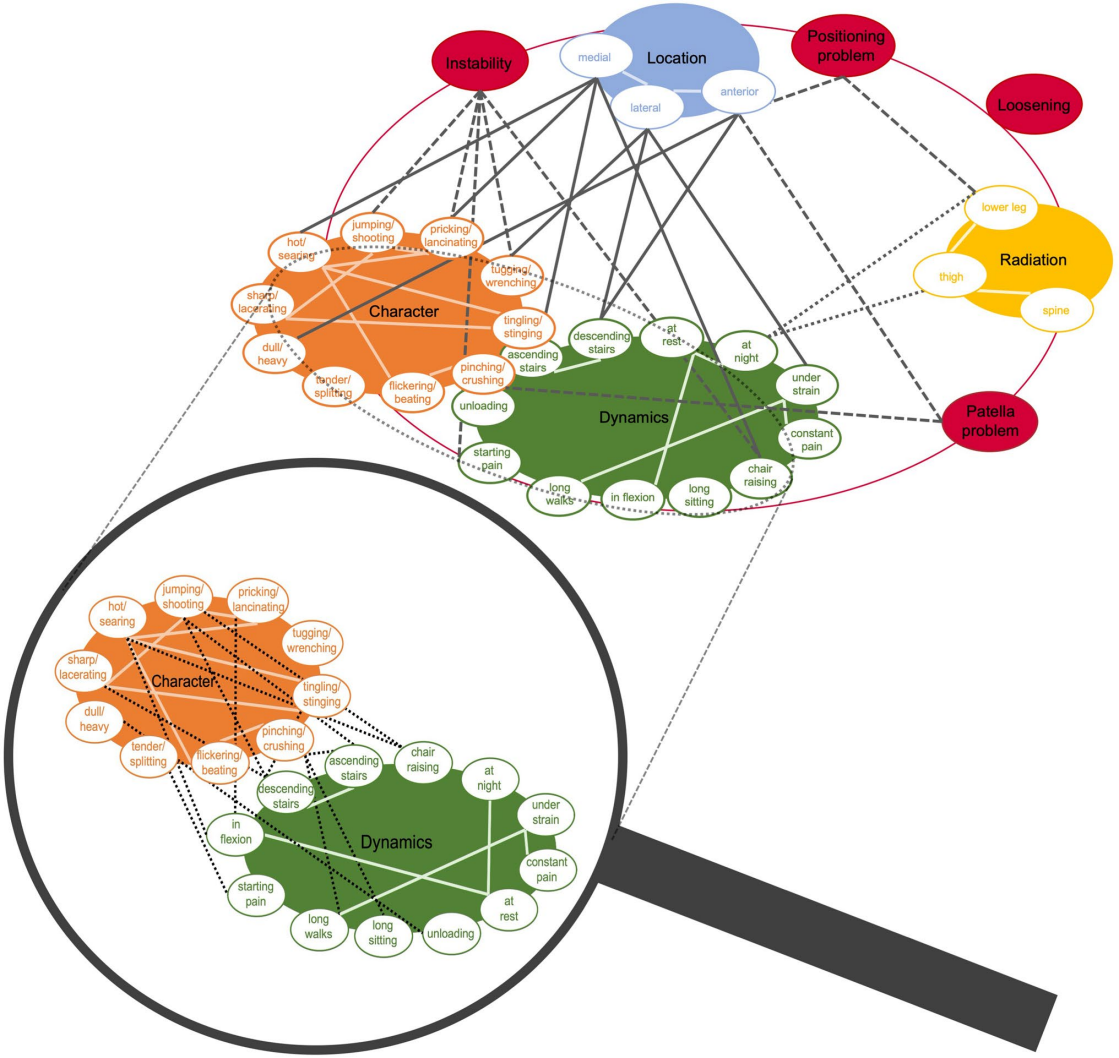
Illustration of pain patterns according to positive Spearman’s correlations among various pain characteristics and pathologies. E.g. instability correlates significantly with jumping/shooting, pricking/lancinating and tugging/wrenching pain character aggravated by chair raising or starting. Magnifier shows correlations between pain characters and dynamics. Reprinted with permission [25]

In 2020, this study group was able for the first time to identify pain characteristics in unhappy patients after TKA and link these to specific underlying pathologies. Based on this, pain patterns were found [25] (Fig. 1). However, objective radiological findings, such as TKA component positioning, were not collected in this study, which constitutes a major limitation.

Over the past 10 years, SPECT/CT has become an increasingly recognized and appreciated diagnostic imaging modality in patients with pain after TKA. A considerable number of studies have been published proving its beneficial clinical use in establishing the diagnosis and providing guidance for further treatment [1, 11, 12, 14, 31]. SPECT/CT allows a combined assessment of structural, mechanical, and functional information [10]. In 2016, a retrospective study was performed on a series of 37 patients after bilateral TKA to evaluate the differences of bone tracer uptake (BTU) in symptomatic and asymptomatic knees after bilateral TKA and identify typical BTU patterns with regards to TKA component position and alignment. The authors could show a significant correlation of TKA component position and BTU and identified typical BTU patterns in symptomatic and asymptomatic knees [2].

To date, there exists no study that links findings of SPECT/CT to specific pain patterns in patients with pain after TKA. The more clinical and radiological information about a patient with pain after TKA is included in the assessment, the more reliable and sustainable the advice regarding TKA revision can be [34].

Therefore, the primary aim of this study was to assess the position of TKA components and evaluate BTU using pre-revision SPECT/CT and correlate these findings with previously published pain patterns in painful patients after TKA. It was hypothesized that specific TKA component positioning and BTU patterns can be correlated with recently identified pain patterns.

## Materials and methods

**Fig. 2:**
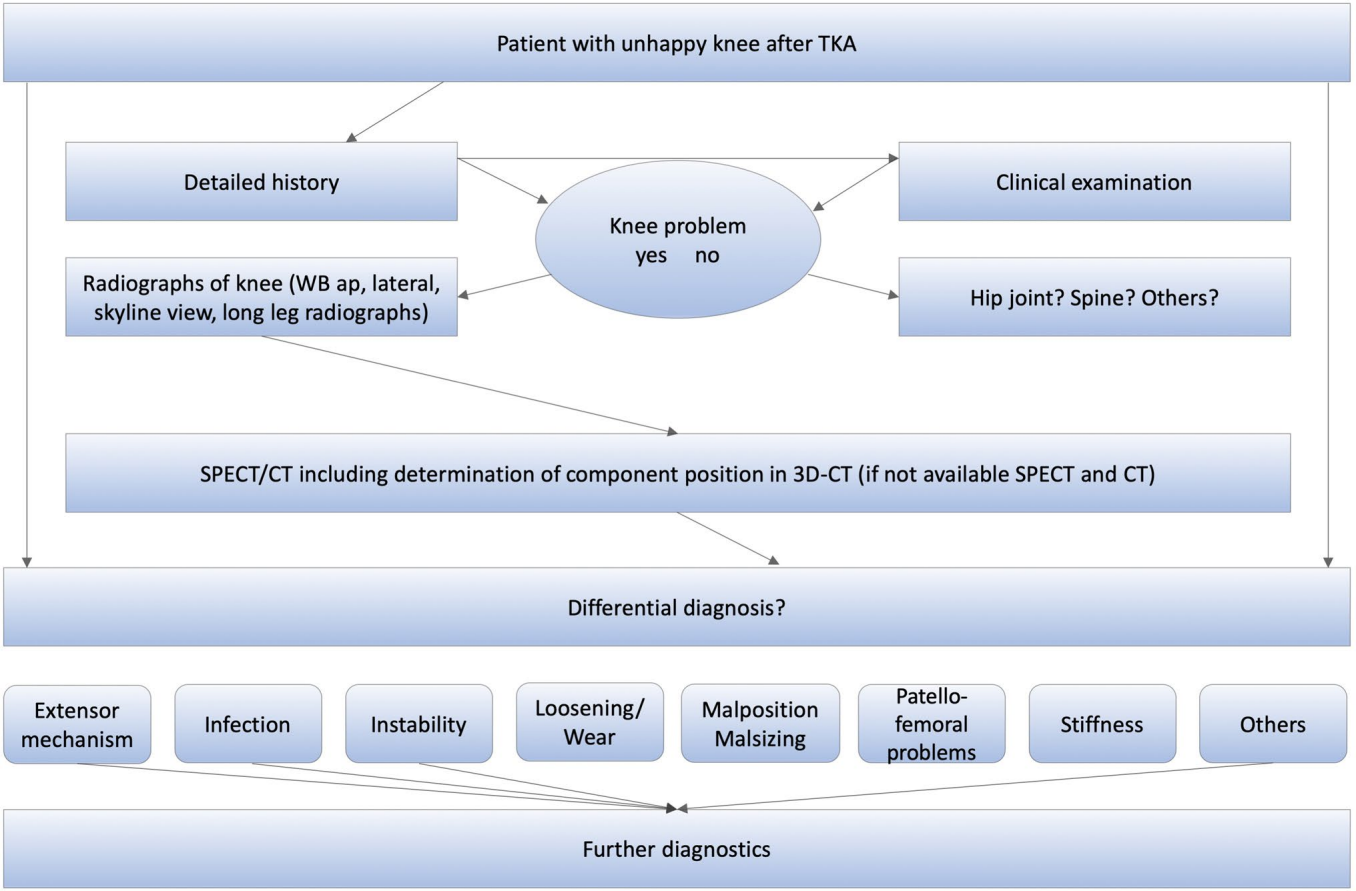
The “Bruderholz” standardized diagnostic algorithm for patients with pain after total knee arthroplasty. WB, weight bearing; SPECT/CT, single photon emission computed tomography/computer tomography. Reprinted with permission [25]

This study was approved by the local ethical committee (2017-02048) and was performed in accordance with the ethical standards of the responsible committee and with the guidelines of the Helsinki Declaration of 1975, as revised in 2008. A written informed consent was signed by every patient. A consecutive number of 83 patients, who underwent primary TKA from 1993 to 2017 and complained about unilateral persistent knee pain and who underwent a revision surgery after completing the diagnostic algorithm including SPECT/CT were prospectively collected and then included in this retrospective cohort study. The cohort is mostly consistent (83 of initially 97 patients) with that in the previously published study on TKA pain patterns [25]. Data were prospectively collected from a specialized knee centre in which the patients presented between 2012 and 2017 due to persistent pain after primary TKA. All patients followed a standardized diagnostic algorithm (Fig. 2) including detailed clinical examination, standardized (anterior–posterior and lateral weight bearing, patellar skyline view) and stress radiographs (anterior–posterior projection with full extension and 30° flexion for varus/valgus laxity; lateral projection in 15° and 90° flexion for anterior/posterior laxity using a Telos device with 15 kp) and 99 m-Tc-HDP-SPECT/CT. At the end of the standardized diagnostic process, the patient's pain was linked to one or more of the pathologies listed in Table 1, which set the indication for the proposed revision surgery. Patients, who have suffered a trauma, underwent revision surgery in other hospitals between primary TKA and presentation at our knee centre, periprosthetic joint infection, patients with exclusively neuropathic pain or not fully completed SPECT/CT protocol were excluded from this study (*N* = 46).

**Table 1:** *Independent* variables: Characteristics of total knee arthroplasty (TKA) and reasons/pathologies responsible for pain. *Dependent* variables: Four characteristics of pain

Characteristics TKA	
Cemented	Tibial, femoral
Uncemented	Tibial, femoral
Cruciate-retaining	
Posterior-stabilized	
Type of polyethylene insert	Mobile-/fixed bearing
Patella resurfaced	
Sagittal degree of congruence between femoral and tibial component	In extension, in 90° flexion

Reasons for revision/pathologies	
Instability	Flexion; anterior–posterior; medial; lateral; multidirectional
Loosening	Tibial, femoral
Position of components	Internal/external rotation of tibial and/or femoral component
	Flexion/extension of femoral component
	Anterior/posterior slope of tibial component
	Varus/valgus of tibial and/or femoral component
	Size: Over-/undersize
Patella problem	Baja; alta; overloading; maltracking; osteoarthritis
Irritation of iliotibial tract	
Arthrofibrosis	
Irritation of infrapatellar branch of saphenous nerve	
Wear of PE tibial inserts	

**Characteristics of pain**	
Character of pain	Flickering/beating, jumping/shooting, pricking/lancinating, sharp/lacerating, pinching/crushing, tugging/wrenching, hot/searing, tingling/stinging, dull/heavy, tender/splitting
Dynamics of pain	At rest, at night, under strain, ascending stairs, descending stairs, unloading, starting pain, long walks, uneven surfaces, walking downhill, full flexion, full extension, long sitting, long standing, chair raising, constant pain
Location of pain	Anterior, posterior, medial, lateral
Radiation	Lower leg, thigh, spine

*PE* polyethylene

### Data collection

Within the framework of the consultations at the knee centre, all the variables listed in Table 1 were described and documented in a consultation report by one expert knee surgeon (senior orthopedic consultant) for each patient in a standardized manner. The character of pain was described according to the sensory pain descriptors (dimension 1–10) used in the McGill Pain Questionnaire [27]. Pain dynamics were categorized according to the seven types of Laskin [19] and extended and adapted to the patient cohort. A member of this study group retrospectively collected and evaluated these criteria based on surgery and consultation reports meticulously and published the resulting pain patterns in 2021 (Fig. 1, [25]). Due to varying frequencies of pain characteristics reported by the patients, the N per dimension is not identical. Thereafter, another study group member evaluated all SPECT/CT images and recorded TKA component positioning and BTU.

### Radiological imaging

All patients underwent 99m-Tc- hydroxymethyl diphosphonate (HDP) SPECT/CT imaging following a standardized and highly reliable protocol [10, 15, 31]. The mean time from primary TKA to SPECT/CT was 2.5 ± 3.0 years (range 0.04–19.7 years, in 20 cases < 12 months). All patients received a commercial 700MBq (18.92mCi) 99m-Tc-HDP injection (Malinckrodt, Wollerau, Switzerland). SPECT/CT was performed using a hybrid system (Symbia T16, Siemens, Erlangen, Germany), which consists of a pair of low-energy, high-resolution collimators and a dual-head gamma camera with an integrated 16-slice CT scanner (collimation of 16 × 0.75 mm). Planar scintigraphic images were taken in the perfusion phase (immediately after injection), the soft tissue phase (1–5 min after injection) and the delayed metabolic phase (at least 2 h after injection). SPECT/CT was performed with a matrix size of 128 × 128, an angle step of 32, and a time per frame of 25 s 2 h after injection.

### Assessment of TKA position and mechanical alignment

**Fig. 3:**
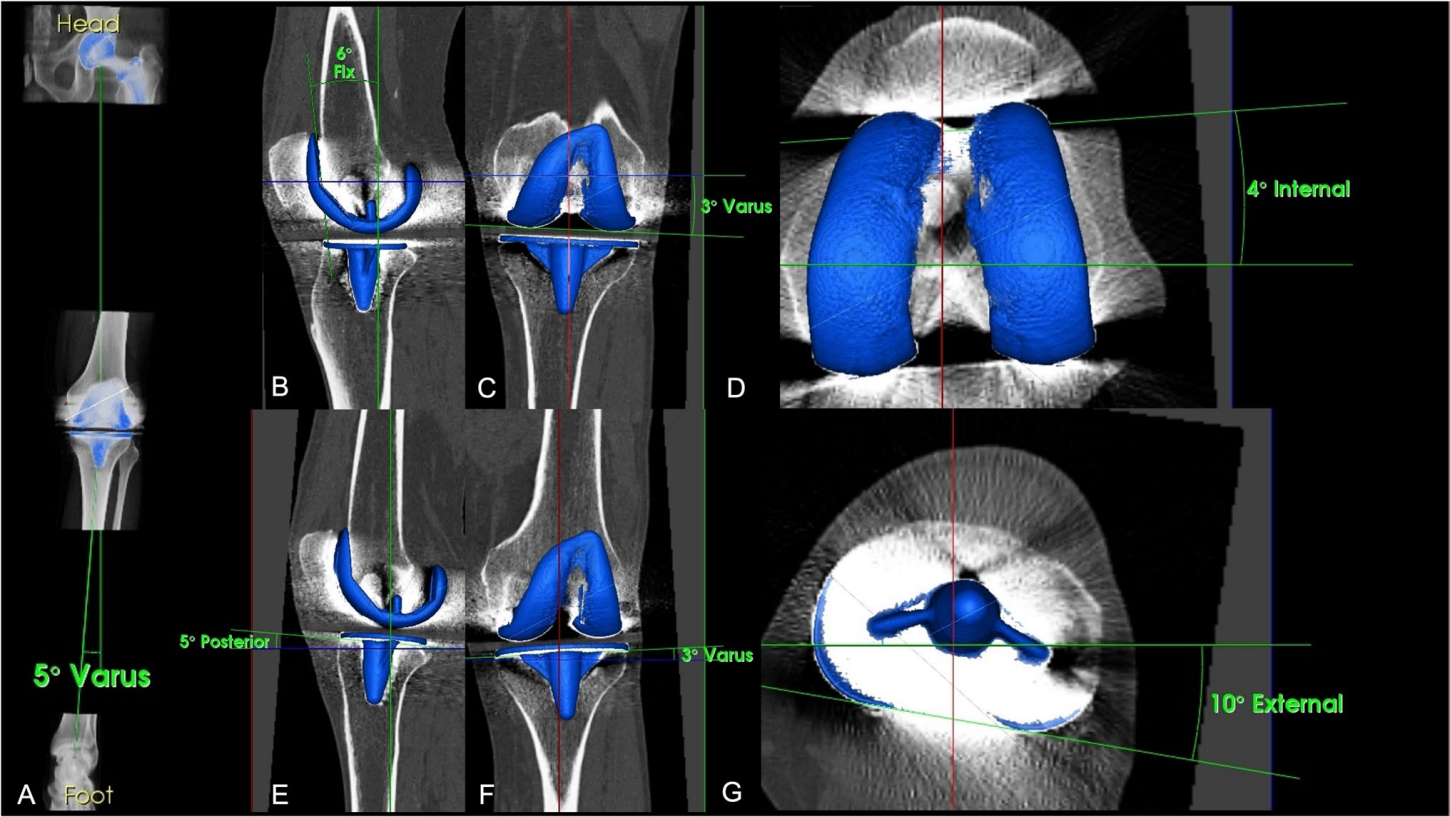
SPECT/CT images of a 74-year-old male patient with left painful total knee arthroplasty (TKA). Mechanical alignment and TKA position were assessed using a customized validated 3D-software “Orthoexpert®”. **a**–**g** show angle measurements of the femoral (**b**–**d**) and tibial (**e**–**g**) component. The increased internal rotation of 4° of the femoral shield (**d**) and varus positioning of 3° of the tibial component (**f**) causes pain in this TKA patient

Mechanical alignment and TKA position were assessed using a customized validated 3D-software which has been proven highly accurate (Fig. 3) [10, 15, 31]. For assessment of mechanical alignment, reconstructed images were displayed in orthogonal axial, coronal, and sagittal planes. Coronal (valgus–varus), rotational (internal–external rotation), and sagittal (flexion–extension, antero-posterior slope) TKA component position were measured in relation to standardized landmarks [31].

For statistical analysis, angle cut-off values had to be determined to distinguish between conspicuous and inconspicuous. Based on the existing literature, the following values were defined as conspicuous [2–4, 6, 8, 9, 17, 36]: Femoral varus/valgus > 2°, flexion/extension > 10°/ < 0° and external/internal rotation > 10°/ > 5°. Tibial varus/valgus > 2°, posterior/anterior slope > 10°(CR), > 5° (PS)/ < 0° (CR, PS) and external/internal rotation > 10°/ > 5°. Tibiofemoral varus/valgus > 2°.

### Measurement of BTU

**Fig. 4:**
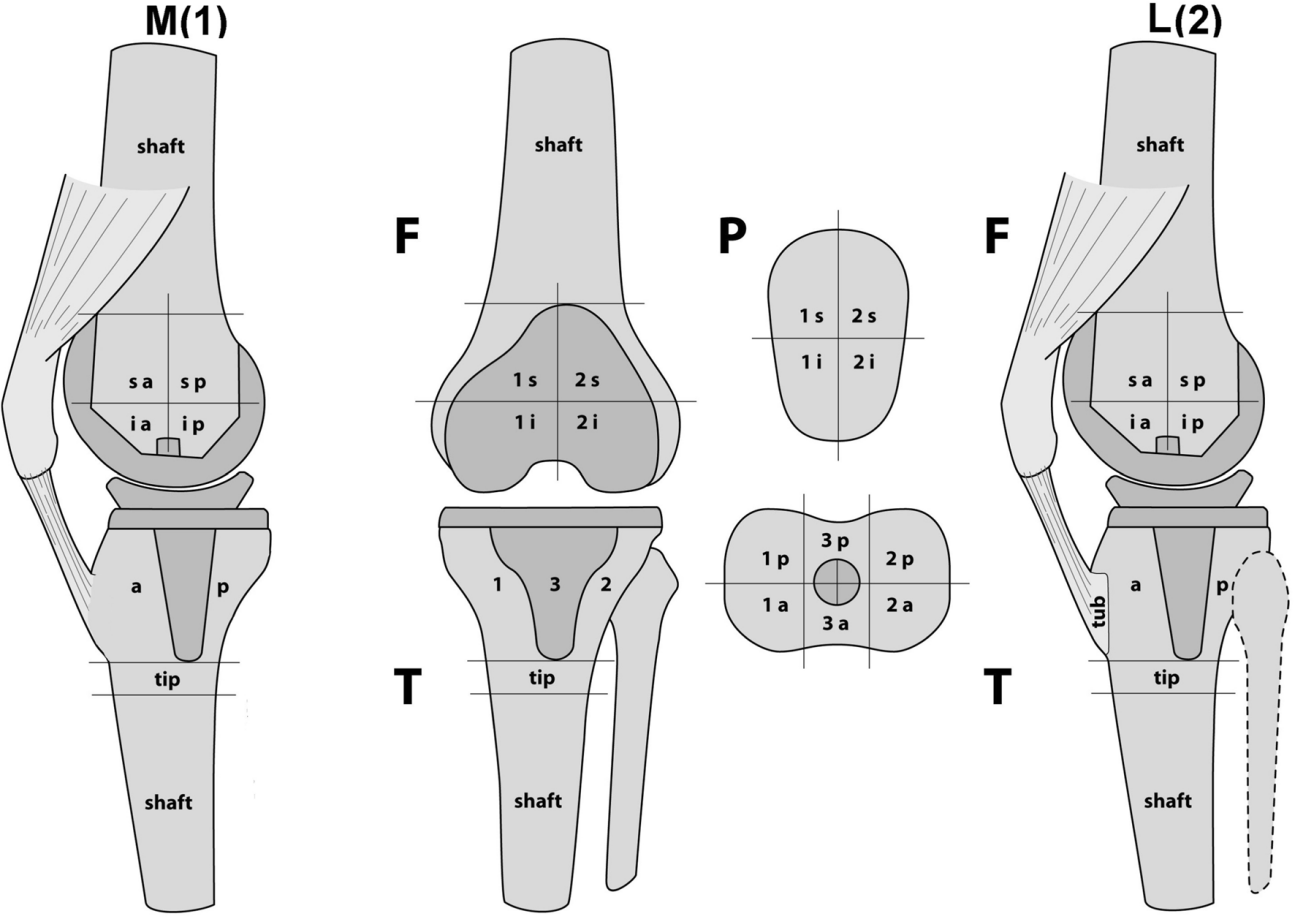
Standardized and validated scheme for localisation of bone tracer uptake after total knee arthroplasty. Reprinted with permission [12]

For BTU, anatomically precise localization and quantification were recorded on the basis of a validated standardized localization scheme [11, 28, 31]. This localisation scheme (1 = medial, 2 = lateral and 3 = central) consisted of eight femoral (f-1sa, f-1ia, f-1sp, f-1ip, f-2sa, f-2ia, f-2sp, f-2ip), eight patellar (p-1sa, p-1sp, p-1ia,p-1ip, p-2sa, p-2sp, p-2ia,p-2ip), and 18 tibial zones (t-1astem, t-1atip, t-1atray, t-1pstem, t-1ptip, t-1ptray, t-2astem, t-2atip, t-2atray, t-2pstem, t-2ptip, t-2ptray, t-3astem, t-3atip, t-3atray, t-3pstem, t-3ptip, t-3ptray). It was used to accurately map the examined BTU volume in each anatomical area of interest (Fig. 4). Mean BTU values (mean ± standard deviation, median, and range) for each area of the localization scheme were recorded and normalized values calculated. For normalization, a specific area within the distal femoral shaft was used as reference region for all zones to obtain ratios of absolute measures. For statistical interpretation, based on factor analysis within femur, patella and tibia, areas were grouped into five major regions (femur total, patella total, tibial tray total, tibia stem and tip lateral, tibia stem and tip medial and central).

### Statistical analysis

Results are presented as means, ranges and standard deviations (SD) or with numbers and percentages. To correlate binary variables, phi coefficients were calculated. Because the cut angles values and some BTU values are not normally distributed, ordinal Spearman correlations (rho) were used for all correlations. Two-sided *p* values < 0.05 were considered significant. All data were analyzed by an independent professional statistician using SPSS Statistics for Windows, version 26.0 (Armonk, NY: IBM Corp, USA). A post hoc analysis using G*Power, version 3.1.9 (University of Kiel, Germany) tested that, for the given *N* = 83, a correlation of rho = 0.28 can be found with a power of 80%. For the number of patients with pain characteristic values (*N* = 59), the same correlation has to be rho = 0.35.

## Results

Patient demographics and TKA characteristics of all 83 patients included are shown in Table 2. Due to the set-up in a specialized knee centre with focus on painful TKA, most of the patients were referred from other surgeons to our clinic. Therefore, primary TKA was performed by a total of 55 different surgeons.

**Table 2:** Patient demographics and TKA characteristics

Variable	Total
Mean patient age at initial consultation knee centre; yrs (SD); range	65.9 (9.5); 46–85
Mean patient age at primary TKA; yrs (SD); range	63.4 (9.2); 42–81
Mean time from primary TKA to initial consultation; yrs (SD); range	2.5 (3.3); 0.1–23
Mean time from primary TKA to revision; yrs (SD); range	3.0 (3.3); 0.3–23
Mean BMI, kg/m^2^ (SD; range)	29.3 (5.4; 18–50)
Female:male, *N* (%)	57 (68.7): 26 (31.3)
Indication primary TKA	
Primary osteoarthritis, *N* (%)	37 (44.6)
Secondary osteoarthritis, N (%)	46 (55.4)
Side, left:right, *N* (%)	34 (59.0): 49(41.0)
Mean degree of sagittal congruence in extension and flexion (SD; range) (*N* = 62)	0.82 (0.21; 0.38–1.00), 0.52 (0.18; 0.22–1.00)

TKA characteristics Knee system	Fem. cem./uncem	Tib. cem./uncem	PS/CR	Mobile/ fixed bearing	Patella resurfaced yes/no	Total *N* (%)
Attune (Johnson & Johnson)	19/1	20/0	4/16	12/8	4/16	20 (24.1)
P.F.C. Sigma (Johnson & Johnson)	7/5	12/0	3/9	5/7	4/8	12 (14.5)
BalanSys (Mathys)	8/2	10/0	1/9	6/4	0/10	10 (12.1)
Triathlon (Stryker)	7/2	9/0	3/6	1/8	1/8	9 (10.8)
LCS Complete (Johnson & Johnson)	1/7	4/4	1/7	8/0	0/8	8 (9.6)
Innex (Zimmer)	4/0	4/0	2/2	2/2	0/4	4 (4.8)
Persona (Zimmer Biomet)	4/0	4/0	2/2	0/4	1/3	4 (4.8)
E.motion (B. Braun)	3/0	3/0	3/0	3/0	2/1	3 (3.6)
Gemini (Link)	2/0	2/0	1/1	1/1	1/1	2 (2.4)
TC-Plus (Smith&Nephew)	0/2	2/0	0/2	1/1	0/2	2 (2.4)
Vanguard (Zimmer Biomet)	2/0	2/0	0/2	1/1	1/1	2 (2.4)
Others	6/1	7/0	1/6	3/4	3/4	7 (8.5)
Total *N* (%)	63(75.9)/20(24.1)	79(95.2)/4(4.8)	21(25.3)/62(74.7)	43(51.8) /40(48.2)	17 (20.5)/66 (79.5)	83 (100)

*N* = 83

*SD* standard deviation; *TKA* total knee arthroplasty; *Fem* femoral; *Tib* tibial; cem./uncem., (un)cemented; *PS* posterior-stabilized; *CR* cruciate retaining; others (1 × Duracon (Stryker), 1 × HLS (Corin), 1 × Legion (Smith&Nephew), 1 × Natural-Knee II (Zimmer Biomet), 1 × NexGen (Zimmer Biomet), 2 × unknown knee system)

Table 3 provides an overview of the pain generators based on the standardized diagnostics. The majority of patients were diagnosed with more than one underlying pathology (77 patients = 91.6%). Characteristics that occurred in less than four patients (*N* < 4) were excluded from the statistical analysis (*N* total = 19; Tables 3 and 4).

**Table 3:** Frequencies (*N* and %) of pathologies responsible for the reported pain, divided in subgroups

Pathology (group)	Pathology (subgroup)	*N*	%
Instability		43	51.8
	Flexion instability	17	20.5
	Anterior–posterior instability	17	20.5
	Medial instability	7	8.4
	Lateral instability	21	25.3
	Multidirectional instability	4	4.8
Loosening		9	10.8
	Tibial loosening	7	8.4
	Femoral loosening	2	2.4
Components		68	81.9
	Femoral component in flexion	21	25.3
	Femoral component in extension	1	1.2
	Femoral component in int. rotation	18	21.7
	Femoral component in varus	15	18.1
	Femoral component in valgus	6	7.2
	Tibial component in posterior slope	10	12.0
	Tibial component in anterior slope	4	4.8
	Tibial component in int. rotation	5	6.0
	Tibial component in ext. rotation	11	13.3
	Tibial component in valgus	4	4.8
	Tibial component in varus	20	24.1
	Tibiofemoral in valgus	4	4.8
	Tibiofemoral in varus	18	21.7
Patella problems		47	56.6
	Patella baja	10	12.0
	Patella overloading	36	43.4
	Patella maltracking	3	3.6
	Osteoarthritis patella	6	7.2
Other problems		23	27.7
	Irritation of iliotibial tract	7	8.4
	Irritation of infrapatellar branch of saphenous nerve	3	3.6
	Arthrofibrosis	12	14.5
	Wear of PE tibial inserts	3	3.6

The percentages totalled > 100% because some knees had more than one pathology (*N* = 83)

*PE* polyethylene

**Table 4:** Dependent variables: Frequencies (*N* and %) of four characteristics of pain

Character of pain (*N* = 59)	*N*	%	Dynamics of pain (*N* = 71)	*N*	%	Location of pain (*N* = 83)	*N*	%	Radiation (*N* = 72)	*N*	%
Flickering/beating	7	12.3	At rest	16	21.1	Anterior	76	92.7	Lower leg	11	15.3
Jumping/shooting	16	27.1	At night	20	26.3	Posterior	3	3.6	Thigh	13	18.1
Pricking/lancinating	28	47.5	Under strain	21	29.6	Medial	52	63.4	Spine	7	9.7
Sharp/lacerating	6	10.2	Ascending stairs	28	39.4	Lateral	54	65.9			
Pinching/crushing	22	37.3	Descending stairs	37	52.1						
Tugging/wrenching	15	25.4	Unloading	10	13.5						
Hot/searing	18	30.5	Starting pain	10	13.2						
Tingling/stinging	11	18.6	Long walks	11	15.5						
Dull/heavy	23	39.0	Uneven surfaces	4	5.6						
Tender/splitting	9	15.5	Walking downhill	8	11.3						
			Full flexion	11	15.5						
			Full extension	3	4.2						
			Long sitting	7	9.9						
			Long standing	3	4.2						
			Chair raising	6	8.5						
			Constant pain	4	5.3						

The percentages totalled > 100%, because some knees had more than one pain character, dynamics, location or radiation

The frequencies of all pain characteristics are shown in Table 4 and correspond to the findings of the preceding study [25]. Most patients described more than one dimension of each pain characteristic.

Measurements of TKA component position in 3D reconstructed CT and BTU in SPECT/CT are shown in Tables 5 and 6. The greatest variation in component positioning was found on the femur in the sagittal plane (1° extension, 19° flexion) and on the tibia in the axial plane (11° internal rotation, 19° external rotation). The highest BTU was found in the supero-lateral areas of the femur and patella, and in the postero-lateral tibial tray.

**Table 5:** Measurements of total knee arthroplasty component position in 3D reconstructed CT scans

Variable (*N* = 83)	Mean	SD	Med	Min	Max
Femur flexion ( +)/extension (−)	7.82	± 4.31	7	− 1	19
Femur external ( +)/internal rotation (−)	− 3.11	± 3.01	− 3	− 11	5
Femur varus ( +)/valgus (−)	0.34	± 2.12	0	− 5	7
Slope anterior ( +)/posterior (−)	− 4.08	± 3.17	− 4	− 12	3
Tibia external ( +)/internal rotation (−)	3.99	± 6.17	3	− 11	19
Tibia varus ( +)/valgus (−)	1.34	± 1.82	1	− 4	5
Tibiofemoral angle varus ( +)/valgus (−)	1.18	± 3.97	1	− 12	17

*SD* standard deviation; *Med* Median; *Min* Minimum; *Max* Maximum

**Table 6:** SPECT/CT mean bone tracer uptake values and relevant dimensions marked in grey according to factor analysis; 1, medial; 2, lateral; 3, central; i, inferior; s, superior; a, anterior; p, posterior

*N* = 83	Mean	Median	Std. Deviation	Minimum	Maximum
Femur 1ia	0.76	0.68	0.37	0.32	2.34
Femur 1ip	0.95	0.83	0.54	0.30	4.05
Femur 1sa	1.04	0.98	0.36	0.30	2.59
Femur 1sp	1.13	1.07	0.49	0.22	2.75
Femur 1 total	0.97	0.94	0.38	0.30	2.49
Femur 2ia	0.82	0.73	0.38	0.31	2.27
Femur 2ip	1.01	0.91	0.43	0.31	2.42
Femur 2sa	1.22	1.20	0.39	0.42	2.70
Femur 2sp	1.14	1.07	0.54	0.14	2.83
Femur 2 total	1.05	1.02	0.37	0.30	2.22
Femur total	1.01	0.97	0.35	0.30	2.08
Patella 1ia	1.48	1.12	1.10	0.23	6.54
Patella 1ip	1.94	1.77	1.19	0.33	6.40
Patella 1sa	2.27	1.65	1.74	0.21	8.98
Patella 1sp	2.40	2.04	1.59	0.32	9.58
Patella 1 total	2.02	1.60	1.26	0.40	6.44
Patella 2ia	1.58	1.20	1.71	0.13	13.90
Patella 2ip	2.31	1.63	1.95	0.27	11.98
Patella 2sa	2.63	1.85	2.94	0.30	23.56
Patella 2sp	2.70	2.18	1.93	0.39	11.35
Patella 2 total	2.31	1.73	1.98	0.54	15.20
Patella total	2.17	1.83	1.53	0.51	10.82
Tibia 1a.tray	1.75	1.48	0.95	0.49	4.63
Tibia 1p.tray	1.82	1.70	0.78	0.60	4.35
Tibia 1 tray total	1.79	1.59	0.80	0.56	4.25
Tibia 2a.tray	1.94	1.90	0.83	0.76	4.36
Tibia 2p.tray	1.99	1.78	0.89	0.80	6.53
Tibia 2 tray total	1.97	1.77	0.79	0.80	4.90
Tibia 3a.tray	1.41	1.29	0.59	0.46	3.58
Tibia 3p.tray	1.48	1.36	0.69	0.37	4.28
Tibia 3 tray total	1.45	1.44	0.57	0.43	3.17
Tibia tray total	1.73	1.59	0.66	0.62	3.75
Tibia 1a.stem	0.97	0.85	0.80	0.19	6.77
Tibia 1p.stem	0.93	0.79	0.73	0.10	6.09
Tibia 2a.stem	1.21	1.08	0.62	0.15	4.12
Tibia 2p.stem	1.15	1.00	0.61	0.17	3.98
Tibia 3a.stem	1.62	1.47	0.60	0.57	3.69
Tibia 3p.stem	1.18	1.02	0.71	0.23	6.06
Tibia 1a.tip	0.49	0.44	0.37	0.11	3.07
Tibia 1p.tip	0.42	0.32	0.35	0.07	2.75
Tibia 2a.tip	0.75	0.63	0.50	0.06	2.87
Tibia 2p.tip	0.57	0.50	0.29	0.13	1.60
Tibia 3a.tip	1.48	1.40	0.66	0.44	5.58
Tibia 3p.tip	0.82	0.74	0.51	0.21	4.68
Tibia stem and tip 1 total	0.70	0.61	0.54	0.12	4.67
Tibia stem and tip 2 total	0.92	0.85	0.45	0.13	3.11
Tibia stem and tip 3 total	1.27	1.19	0.56	0.40	5.00
Tibia stem and tip 1 and 3 total	0.99	0.92	0.53	0.26	4.84
Tibia stem and tip total	0.96	0.93	0.47	0.21	4.26
Tibia total	1.22	1.16	0.47	0.35	3.81
Total (fem./tib./pat.)	1.38	1.27	0.63	0.51	4.54

Numerous significant correlations were found between various pain and patients characteristics and SPECT/CT findings including TKA component positioning (Tables 7 and 8).

**Table 7:** Correlation of TKA pain characteristics with SPECT/CT findings according to Spearman’s rho

		Character (*N* = 59)										Location (*N* = 82)			Dynamics (*N* = 71)													Radiation (*N* = 72)		
		Hot/ searing	Jumping/ shooting	Tingling/ stinging	Pricking/ lancinating	Tugging/ wrenching	Pinching/ crushing	Flickering/ beating	Tender/ splitting	Dull/ heavy	Sharp/ lacerating	Anterior	Medial	Lateral	At rest	At night	Under strain	Ascending stairs	Descending stairs	Unloading	Starting pain	Long walks	Walking downhill	In flexion	Long sitting	Chair raising	Constant pain	Lower leg	Thigh	Spine
TKA component positioning	Femur flexion	− 0.14	− 0.18	− 0.08	− 0.19	− 0.18	0.2	0.09	0.31*	0.07	− 0.08	0.07	− 0.07	0	− 0.24*	− 0.16	− 0.09	0.07	0.09	0.03	0.04	0.08	− 0.09	0.02	0.12	− 0.28*	0	0.07	− 0.14	0.13
	Femur internal rotation	0.1	− 0.01	0.11	0.03	0.22	0.04	− 0.01	− 0.13	0.13	0.03	− 0.04	0.14	0.21	− 0.09	− 0.13	0.01	0.16	0.1	0.12	− 0.06	0.05	0.03	− 0.05	0.04	0.01	− 0.18	0.02	− 0.1	− 0.01
	Femur valgus	0.02	− 0.02	− 0.21	0.02	− 0.02	0.01	0.03	0.08	− 0.18	− 0.09	− 0.09	0.05	0.04	− 0.16	0.23*	0.03	0.01	− 0.03	− 0.12	0.02	− 0.1	0.04	− 0.03	− 0.11	0.02	0.39***	− 0.14	0.16	0.03
	Femur varus	− 0.01	− 0.05	0.26*	0.07	0.03	0.03	0	− 0.19	0.26*	0.19	− 0.02	0.07	0.02	0.13	− 0.18	0.06	0.1	0.27*	0.24*	− 0.09	0.12	− 0.24*	0.07	0.26*	− 0.16	− 0.2	− 0.1	− 0.15	0.14
	Tibia post. slope	0.01	− 0.13	0.07	− 0.23	0.22	0.19	− 0.01	− 0.17	− 0.02	0.17	0.19	0.08	0.01	0.06	0.11	− 0.12	− 0.07	− 0.16	− 0.02	− 0.08	0.11	− 0.05	0.18	− 0.13	0.01	0.23*	0.04	0.1	0.16
	Tibia ant. slope	0.02	0.05	0.02	0.09	− 0.22	− 0.24	− 0.11	− 0.01	− 0.04	0	− 0.16	− 0.15	− 0.17	0.03	− 0.03	0.02	− 0.04	0.09	0.03	− 0.07	− 0.15	0.01	− 0.06	− 0.07	0.07	− 0.08	− 0.11	− 0.09	− 0.23
	Tibia internal rotation	− 0.06	− 0.22	0.06	0.19	0.3*	− 0.11	− 0.09	0.24	− 0.03	0.1	0.09	− 0.04	0.1	0.09	0.16	− 0.24*	− 0.08	0.12	0.25*	0.17	− 0.03	0	0.02	0.1	− 0.11	− 0.03	0.08	0.2	0.32**
	Tibia external rotation	0.15	− 0.1	− 0.09	− 0.1	− 0.05	0.03	− 0.03	− 0.2	− 0.01	0.04	− 0.2	0.06	0.01	− 0.01	− 0.2	0.17	0.14	0.01	− 0.07	− 0.13	0.04	− 0.11	0.1	− 0.24*	0.07	− 0.07	− 0.03	− 0.07	− 0.11
	Tibia valgus	0.01	0.06	− 0.09	− 0.12	0.08	− 0.04	− 0.16	− 0.18	− 0.06	0	0.1	− 0.16	− 0.1	0.12	0.15	0.05	− 0.29*	− 0.1	− 0.02	− 0.02	− 0.15	0.15	0.1	− 0.12	− 0.11	0.11	0.08	0.17	− 0.12
	Tibia varus	− 0.13	− 0.03	− 0.07	0.17	− 0.18	0	0.04	0.08	− 0.05	0.02	− 0.01	0.14	0.04	− 0.11	− 0.24*	0.21	0.17	0.1	− 0.06	0.26*	0.15	− 0.17	− 0.19	0.12	− 0.09	0.07	− 0.2	− 0.21	− 0.15
	Tibiofemoral valgus	− 0.16	− 0.06	− 0.28*	− 0.24	− 0.02	0.11	− 0.09	0.09	− 0.28*	− 0.16	0.01	− 0.06	− 0.15	− 0.11	0.28*	− 0.07	− 0.11	− 0.19	− 0.03	− 0.07	− 0.01	0.24*	− 0.02	− 0.1	− 0.01	0.23*	− 0.07	0.01	− 0.11
	Tibiofemoral varus	− 0.17	− 0.04	0.12	0.2	− 0.02	0.02	0.05	0.01	0.19	0.19	− 0.02	0.07	0.06	0	− 0.18	0.11	0.16	0.23	0.13	0.12	0	− 0.27*	− 0.09	0.04	− 0.14	− 0.15	0.01	− 0.1	0.19
SPECT/CT BTU	Femur total	0.12	− 0.06	− 0.05	0.07	0.21	− 0.1	0.03	0.05	− 0.08	− 0.1	0.07	0.12	0.14	0.09	0.08	− 0.01	0.06	− 0.08	− 0.12	0.06	0.03	0.14	0.13	− 0.18	0.1	− 0.08	0.04	0.06	− 0.01
	Patella total	0.05	0.13	− 0.18	− 0.17	− 0.02	− 0.03	− 0.12	0.02	− 0.11	− 0.01	− 0.03	0.13	0.09	0.12	0.21	− 0.04	0.1	− 0.05	− 0.08	0.13	− 0.16	0.05	0.11	− 0.11	0.16	− 0.02	0.07	− 0.09	− 0.26*
	Tibia tray total	0.09	0.05	− 0.09	0.08	0.14	− 0.11	− 0.07	− 0.01	− 0.17	− 0.08	0.08	0.03	0.1	0.03	0.07	− 0.03	0.11	− 0.15	0.06	0.17	− 0.03	− 0.01	0.31**	− 0.06	0.04	− 0.15	0.03	0.03	− 0.15
	Tibia stem and tip lateral	− 0.08	− 0.01	− 0.15	0.03	0.11	0.11	− 0.27*	0.03	− 0.26*	0.06	0.13	0.14	0.32**	0.19	0.13	0.18	− 0.06	− 0.08	− 0.16	0.06	0.02	− 0.03	0.12	− 0.2	0.07	0.26*	0.01	0.06	− 0.16
	Tibia stem and tip medial &central	0.06	− 0.01	− 0.18	0.04	0.17	− 0.01	− 0.17	− 0.02	− 0.24	− 0.18	0.09	0.12	0.2	0.17	0.07	− 0.01	0.11	− 0.16	− 0.15	− 0.01	− 0.05	− 0.03	0.2	− 0.13	0.21	0.06	− 0.15	− 0.08	− 0.16

*BTU* bone tracer uptake

***p < 0.001, **p < 0.01, *p < 0.05

**Table 8:** Correlation of patient and TKA characteristics and pathologies with SPECT/CT findings according to Spearman’s rho

*N* = 83		Gender (female–male)	BMI	Fem. cem.–uncem	Tib. cem.–uncem	Mobile bearing–fixed bearing	PS-CR	Patella resurf.–unresurf	Insta-bility	Loosening	Patella problem	Others
TKA component positioning	Femur flexion	− 0.04	− 0.1	0.22*	0.23*	− 0.13	0.11	− 0.14	− 0.24*	0.11	0.23*	0
	Femur internal rotation	0.14	− 0.01	0.1	0	− 0.17	0.13	− 0.08	0.11	0.15	− 0.01	− 0.18
	Femur valgus	0.11	− 0.13	0.04	0.08	− 0.25*	0	0.14	0.11	− 0.05	− 0.01	0.07
	Femur varus	0	0.3**	− 0.04	− 0.09	0.05	0.02	− 0.22*	− 0.19	0.02	− 0.07	− 0.06
	Tibia post. slope	0.13	0.13	0.13	0.07	0.09	− 0.21	0.09	− 0.04	0.1	0.07	− 0.03
	Tibia ant. slope	0.02	− 0.16	− 0.07	− 0.08	− 0.17	0.23*	− 0.04	− 0.06	− 0.13	− 0.02	0.1
	Tibia internal rotation	− 0.13	0.15	− 0.12	0.02	− 0.21	− 0.09	0	− 0.13	− 0.03	0.01	0.02
	Tibia external rotation	0.18	0.01	− 0.13	− 0.12	− 0.06	− 0.19	0.02	− 0.04	− 0.04	− 0.09	0
	Tibia valgus	− 0.02	− 0.11	− 0.2	0.08	− 0.19	− 0.11	− 0.08	− 0.05	− 0.12	− 0.08	0.39***^a^
	Tibia varus	− 0.06	0.23*	− 0.08	− 0.15	0.01	− 0.23*	− 0.04	0.09	0.16	− 0.01	− 0.18
	Tibiofemoral valgus	− 0.02	− 0.22*	0.09	0.05	− 0.17	0	0.03	− 0.08	− 0.05	− 0.03	0.18
	Tibiofemoral varus	0.11	0.39***	− 0.18	− 0.16	0.01	− 0.05	− 0.09	− 0.08	0.13	− 0.07	− 0.04
SPECT/CT BTU	Femur total	− 0.13	0.02	− 0.05	0	− 0.09	− 0.25*	− 0.04	− 0.18	0.03	0.03	0.13
	Patella total	− 0.3**	0.15	0.16	0.15	0.04	− 0.11	0.2	− 0.05	− 0.17	0.12	− 0.03
	Tibia tray total	− 0.17	0.02	0.05	0.11	0.08	− 0.23*	0.15	− 0.07	0.18	− 0.02	0.08
	Tibia stem and tip lateral	− 0.14	0.19	0.01	0.19	0.07	− 0.28*	− 0.06	− 0.02	0	0.15	0.06
	Tibia stem and tip medial and central	− 0.01	0.12	0	0.12	− 0.02	− 0.25*	0.07	0.03	0.12	− 0.09	0.1

*BTU* bone tracer uptake; *BMI* body mass index; *fem* femoral; *tib* tibial; *(un)cem.* (un)cemented; *PS* posterior-stabilized; *CR* cruciate-retaining; *(un)resurf.* (un)resurfaced.

***p < 0.001, **p < 0.01, *p < 0.05

^a^Arthrofibrosis

**Fig. 5:**
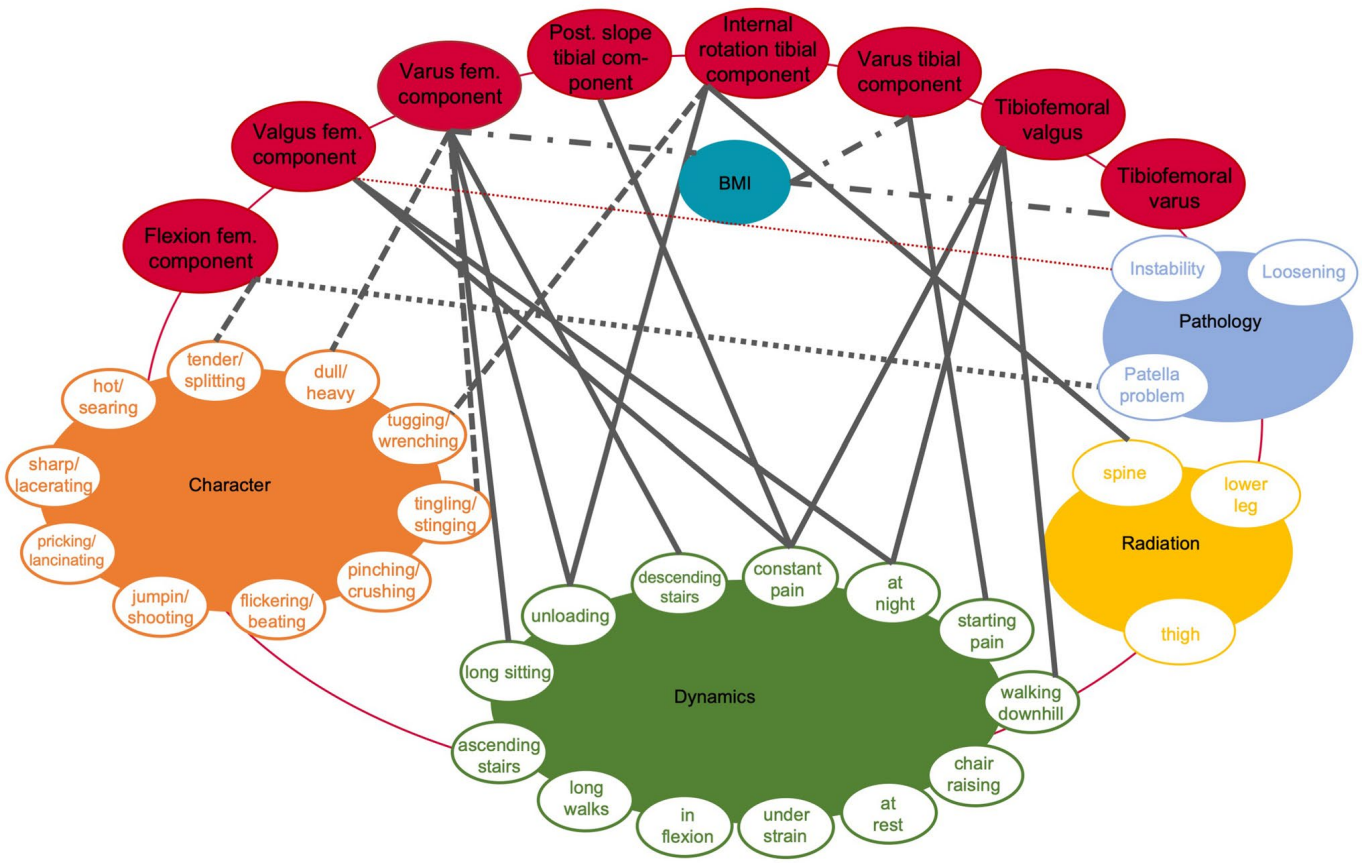
Illustration of pain patterns according to positive Spearman’s correlations with TKA component positioning and pathologies. E.g. More flexion in the femoral component correlates with tender/splitting pain character and patella-related pathologies. Fine, dotted red line: association not significant. *Fem* femoral; *BMI* body mass index

Based on the correlations found the following patterns (*P1–P9*) were identified (Fig. 5):

- *P1:* More flexion in the femoral TKA component is associated with *tender/splitting* pain and patella-related pathologies (*p* < 0.05).
- *P2:* More valgus in the femoral TKA component is associated with constant pain (*p* < 0.001), in particular at night (*p* < 0.05), and instability-related pathologies.
- *P3:* More varus in the femoral TKA component is associated with *dull/heavy* and *tingling/stinging* pain during descending stairs, unloading and long sitting (*p* < 0.05) in patients with high BMI (*p* < 0.01) and unresurfaced patella (*p* < 0.05).
- *P4:* More internal rotation of the tibial TKA component is associated with *tugging/wrenching* pain during unloading (*p* < 0.05) and radiation to the spine (*p* < 0.01).
- *P5:* More varus in the tibial TKA component is associated with starting pain (p < 0.05) in patients with high BMI *(p* < *0.05)*.
- *P6:* More posterior slope in the tibial TKA component is associated with constant pain (*p* < 0.05).
- *P7:* More tibiofemoral valgus alignment is associated with constant pain, in particular at night, and by walking downhill and with low BMI (*p* < 0.05).
- *P8:* More tibiofemoral varus alignment is associated with patients with high BMI (*p* < 0.001).
- *P9:* Increased BTU laterally at the stem and tip of the tibial component is associated with constant pain (*p* < 0.05), whereas increased BTU in the area of the tray is associated with patients with pain aggravation in flexion (*p* < 0.01).

## Discussion

The principal finding of this study is the assignment of pain patterns to TKA component positioning in painful patients after TKA. These patterns were identified with regards to pain character, location, dynamics and radiation and linked to specific component positions in all radiological planes.

The most important findings and implications of this study were the following:

First of all, in this study, component-positioning-related pathologies accounted for the greatest proportion (81.9%) followed by patella-related problems (56.6%) and instability (51.8%). This is in contrast to most register data published in recent years [20–22]. In most registries, aseptic loosening and infection followed by instability lead the list of TKA failures. An explanation for this distribution of frequencies might be the standardized, diagnostic work-up of our patients including routinely performed conventional and stress radiographs as well as 3D-SPECT/CT scans [26]. The latter was used to determine the specific component position in three radiological planes in all patients included. Thus, all possible causes for pain are systematically evaluated and in- or excluded in the course of the diagnostic process. It is, therefore, not surprising, that in most patients (91.6%), more than one pathology was found and TKA component-, instability- and patella-related problems are heading the list [26]. These findings correspond to the results of Hofmann et al. who found also high proportions of component- (54%) or alignment-related (41%) failure modes when malalignment were routinely examined [16]. In 50% of the revision cases, they held two or more reasons responsible for the implant failure [16].

Second, correlations were found between TKA component positioning, pain and TKA characteristics and other pathologies. More flexion in the femoral component was significantly associated with *tender/splitting* pain and patella-related pathologies (*P1*). More valgus in the femoral component and in the tibiofemoral alignment correlated with constant pain, in particular at night (*P2, P7*), whereas more varus in the femoral component correlated with *dull/heavy* and *tingling/stinging* pain during long sitting, descending stairs and by unloading in patients with unresurfaced patella (*P3*). Femoral component positioning in valgus (*P2*) and internal rotation was associated with instability-related pathologies, however, not significantly. More internal rotation of the tibial component correlated with *tugging/wrenching* pain during unloading and radiation to the spine (*P4*), whereas increased posterior slope in the tibial component caused constant pain (*P6*). Interestingly, a strong correlation was found between arthrofibrosis (other problems) and valgus positioning of the tibial component (Table 8). A clear explanation for this cannot be given. Although it is known that correct positioning in the sagittal plane of the prosthesis is imperative to achieve satisfactory ROM after TKA [24], there is no literature on the influence of component positioning in the coronal plane on arthrofibrosis. One speculation could be that excessive valgus in the tibial component leads to increased polyethylene wear and this might promote arthrofibrosis. Tibiofemoral not only varus alignment, but also varus positioning of the femoral and tibial component itself, correlated with patients with higher BMI (*P3, P5, P8*)—tibiofemoral valgus alignment with lower BMI. The association between obesity and increased risk of varus malalignment post-surgically has been described in literature in the past [7].

These findings play a crucial role in the diagnostics of patients with painful TKA. Component positioning represents a major challenge in this process and often confronts the clinician with the question whether a specific component position is considered as pathological or within the range of the “norm”. It is generally agreed, that TKA component positioning should not be viewed in absolute terms, but in the context of the complaints as a whole within the diagnostic process. However, reference values, which were also used in this study for statistical purposes to distinguish between conspicuous and inconspicuous TKA component positioning, are provided in the literature as follows [2–4, 6, 8, 9, 17, 36]: The femoral TKA component should be positioned with 0° ± 2° varus/valgus towards the mechanical axis. In the sagittal plane 5° ± 3° flexion can be accepted, as approximately 5–7° of flexion are built into the anterior flange of the femoral TKA component. The axial alignment should be between maximally 10° external and 5° internal rotation [2]. The tibial TKA component should be positioned with 0° ± 2° varus/valgus towards the mechanical axis. In the sagittal plane, it is generally aimed for a posterior slope of 5°–7° in a posterior cruciate retaining (CR) TKA and 0°–3° in a posterior cruciate substituting (PS) TKA. The rotational orientation should be set between 10° external and maximally 5° internal rotation. Femoral internal rotation of more than 5° may lead to patellofemoral overloading of the lateral patellar facet and lateral lift-off of the femoral condyle from the polyethylene inlay, which is called mid-flexion instability [2, 4, 8]. This association was seen in our data, too, but below significance level. This might be due to the fact, that the expression of internal rotation of the femoral component was not very strong (median -3°), but the most extreme value was -11°. The same can be applied to the not-significant association of valgus positioning of the femoral component and instability-related complaints. In the sagittal plane, flexion of the femoral TKA component increases the patellofemoral pressure and leads to a “pseudo” patella baja [33]. This correlation in combination with *tender/splitting* pain character was also found in *P1*. Anterior slope of the tibial TKA component may lead to a tight flexion gap and subsequent flexion deficit. Generally, it is aimed for a posterior slope of 0°–7° [3]. Clearly, this depends on the type of TKA implant [3]. In a posterior cruciate retaining (CR) TKA it is aimed for a posterior slope of 5°–7°, in a posterior cruciate substituting (PS) TKA 0°–3° [2]. Thus, this explains the correlation found in this study between anterior slope of the tibial component and PS inserts (Table 8). Tibial internal rotation may lead to patellofemoral maltracking, popliteal tendon impingement and anterior and posterior soft tissue pain [6, 36]. Based on the results of this study, more variables associated with internal tibial rotation can be added: *tugging/wrenching* pain character, aggravation when unloading and radiation to the spine.

However, there is an individual range of TKA component position in each direction, which is accepted by the patient, which we call the “envelope of TKA position” [2]. Awengen et al. postulated, that this envelope may be slightly different between patients. If this envelope is narrow a slight deviation from the optimal TKA position leads to pain after TKA. If this envelope is wide, a rather large deviation does not cause any problems [2].

A significant correlation between TKA component positioning and pain localisation could not be demonstrated in this study. However, this is not surprising as in the previously published pain patterns by Mathis et al., the anatomical location has been proven no being helpful in localizing the underlying problem [25].

Third, highest mean BTU in SPECT/CT was found in supero-lateral areas of the femur and patella, and in the postero-lateral tibial tray. Areas in the patella and around the tibial tray showed almost twice as high mean BTU values than femoral or stem and tip areas of the tibia. Therefore, one could argue that increased BTU at femoral areas and areas around the tibial stem or tip are more specific for identification of pathologies in patients with TKA. These findings are consistent with previous results of Awengen et al. who compared BTU distribution patterns in asymptomatic and symptomatic TKA patients [2]. However, in regards to correlations of BTU with pain or TKA characteristics, the results are less pretentious. Only an agreement between BTU and pain localisation could be shown. However, a finding which has already been described in the past [13]. Furthermore, the results suggested that increased BTU laterally at the stem and tip caused more constant pain, whereas BTU around the area of the tibial tray was increased in patients with pain aggravation in flexion. Men showed significantly higher BTU in the patella compared to women and CR knee systems showed higher BTU in femur and tibia. With regard to the latter, it can be speculated that CR knee systems generally allow for larger antero-posterior translation compared to PS systems based on the increased posterior slope (-12 to 3° in this cohort) [18], thereby generating more stress on the tibia and femur resulting in higher BTU [2, 14].

Several limitations of the present study have to be acknowledged. This was a retrospective series of prospectively collected 83 TKAs assigned to a specialized knee centre, operated by a team of different surgeons from various hospitals. Thus, a prospective study design including only painful TKAs from one surgeon would improve the comparability of the patient significantly. Another limitation is the restriction of underlying pathologies to anatomical and mechanical nature. It is a well-known fact, that psychological and social determinants play an important role in the perception of pain in patients after TKA [29]. However, these factors have not been assessed in this study. A more systematic and multidimensional pain assessment according to international guidelines should be aimed for in a future study.

## Conclusion

The painful TKA remains a challenge for the surgeon [16]. Revision TKA for unexplained knee pain might harm even more. At 2 and 5 years after a TKA revision, pain is still reported three times more frequently than after a primary arthroplasty [32]. Therefore, before revising a TKA, it is inevitable to perform a conscientious and comprehensive clarification and evaluation of all eligible causes of failure. The clinician reviewing patients with a painful TKA should integrate as many variables as possible in the algorithm to reach the diagnosis. The evaluation of the TKA component positioning is an important part of this diagnostic process. The interpretation of the positioning, however, is very complex and often challenging even for experienced surgeons. The results of this study help to place component positioning in the overall context of the "painful knee arthroplasty" including specific pain patterns. Hence, these data support our hypothesis that specific TKA component positioning and BTU patterns can be correlated with typical pain characteristics.

The findings of this study add significant value to the previously published TKA pain patterns and further differentiate the clinical picture of a painful knee after TKA. Knowing these pain patterns to its utmost extent enables a prediction of the cause of the pain to be made as early as possible in the diagnostic process. If the causes of the described complaints are known, a decision for a necessary therapy can be made reliably and sustainably at an early stage before the state of pain becomes chronic.

## Supplementary Information

Below is the link to the electronic supplementary material.

Supplementary file1 (PDF 67 KB)

Supplementary file2 (PDF 67 KB)

Supplementary file3 (PDF 66 KB)

## Abbreviations

TKA: Total knee arthroplasty
SPECT/CT: Single-photon-emission-computed-tomography/computer tomography
BTU: Bone tracer uptake
HDP: Hydroxymethylen diphosphonate
MRI: Magnetic-resonance imaging
P1-9: Pattern 1–9
CR: Cruciate-retaining
PS: Posterior-stabilized
